# Effects of a Reflection-Integrated CICARE Program on Empathy and Clinical Judgment in Nursing Students: A Quasi-Experimental Study

**DOI:** 10.3390/nursrep16090296

**Published:** 2026-08-24

**Authors:** Qingqing Feng, Min Liu, Tang Tang, Lulu Zhang

**Affiliations:** Department of Nursing, Clinical College of Anhui Medical University, Hefei 230031, China; ching@aycc.edu.cn (Q.F.);

**Keywords:** nursing education, CICARE, communication competence, empathy, clinical judgment, reflective learning, undergraduate nursing students, quasi-experimental study, patient-centered care

## Abstract

**Background/Objectives:** Communication training in undergraduate nursing skills courses is often overshadowed by procedural skill acquisition, leaving limited opportunities for students to develop patient-centered communication skills and engage in reflective learning. This study evaluated a reflection-integrated CICARE communication program embedded in a Fundamentals of Nursing skills course and explored students’ learning experiences. **Methods:** A quasi-experimental study with an embedded qualitative component was conducted among second-year undergraduate nursing students in China. The quantitative component used a non-equivalent control group design, while reflective journals from the Intervention Group provided qualitative data on students’ learning experiences. Two intact classes were assigned to the Intervention Group (*n* = 65), and two intact classes were assigned to the Control Group (*n* = 66). The Intervention Group received an 18-week CICARE-based communication program integrated into skills training, including scenario-based role-play, emotional cue recognition, demonstration videos, feedback, and reflective journals. The Control Group received conventional skills training. Outcomes included communication competence, empathy, professional identity, clinical judgment, and course performance. Analysis of covariance was used to compare post-intervention outcomes after adjustment for baseline scores. Reflective journals from the Intervention Group were analyzed thematically. **Results:** After adjustment for baseline scores, the Intervention Group showed significantly higher communication competence than the Control Group (adjusted mean difference = 2.699, 95% CI: 0.108–5.291, *p* = 0.041). Empathy was also higher in the Intervention Group (adjusted mean difference = 3.796, 95% CI: 0.538–7.053, *p* = 0.023). The between-group difference in professional identity was not statistically significant (*p* = 0.073). The Intervention Group achieved higher total clinical judgment scores than the Control Group (31.52 ± 5.50 vs. 27.20 ± 6.45, *p* < 0.001), with significant differences in noticing, interpreting, and reflecting. Course performance was also higher in the Intervention Group. In their reflective journals, students described greater patient-centered awareness, attention to patients’ emotional needs, professional reflection, and motivation for self-directed learning. **Conclusions:** Integrating CICARE-based communication training with reflective learning may strengthen communication competence, empathy, clinical judgment, and course performance in undergraduate nursing skills education. Further studies with longer follow-up are needed to determine whether the observed differences persist over time and whether professional identity and action-oriented clinical responses improve with longer educational exposure. **Registration:** This study was not registered.

## 1. Introduction

Communication competence is a core component of nurses’ professional competence and a fundamental prerequisite for ensuring the quality of nursing care, fostering therapeutic nurse-patient relationships, and promoting patient recovery [1,2]. However, current fundamental nursing skills training curricula place predominant emphasis on technical procedures, with relatively limited attention to the development of communication competence and the humanistic aspects of care [3,4,5]. Consequently, nursing students frequently demonstrate inadequate communication behaviors during clinical skills practice, such as unclear information delivery, inconsistent verbal communication, and insufficient responsiveness to patients’ emotional needs [6,7]. A national survey conducted in 22 hospitals in China found that 17.8% of adverse events involving 1173 nursing interns were related to clinical practice, with poor communication identified as an important contributing factor [8,9].

The CICARE communication model—Connect, Introduce, Communicate, Ask, Respond, and Exit—was originally developed by UCLA Health and has since been implemented in healthcare settings beyond its original institution [10,11]. Characterized by clear, structured, and replicable steps, the model emphasizes proactive communication, emotional responsiveness, and confirmation of information. These features align closely with the repetitive and protocol-driven nature of fundamental nursing skills training [12]. Although existing studies have primarily focused on the application of CICARE among clinical nurses and other healthcare professionals, its use in undergraduate nursing education, especially in skills laboratories, has received less attention [13,14].

At the same time, student-centered educational approaches that integrate structured communication practice, reflection, and experiential learning into nursing skills training have gained increasing attention [15]. Nursing students’ communication experiences, emotional reactions, and situational perceptions during skills practice may shape how communication competence is developed and internalized [16,17,18]. Incorporating reflective journals, scenario-based experiences, and standardized patient feedback offers an opportunity to identify the challenges students encounter and to inform the design of training approaches that align more closely with learners’ needs. These strategies may also support deeper learning and greater engagement during communication training.

Recent international evidence further supports the educational value of combining reflective and experiential strategies in nursing education. Ahmadpour et al. found that narrative writing based on Gibbs’ reflective model improved nursing students’ empathy and communication skills, suggesting that structured reflection may help students translate clinical experiences into interpersonal learning [19]. Similarly, technology-supported communication simulation has been shown to improve communication knowledge, self-efficacy, communication performance, and compassion among nursing students [20]. Recent studies focusing on clinical judgment have also demonstrated the value of simulation and structured reflection in promoting students’ abilities to notice, interpret, respond to, and reflect on clinical situations [21,22]. These findings suggest that reflection, communication practice, and simulation can each contribute to important nursing competencies; however, these educational components have often been examined separately rather than as part of an integrated communication-focused teaching approach.

Despite the increasing use of CICARE in clinical practice, evidence regarding its integration into undergraduate nursing skills training remains limited. Few studies have explored how structured communication models influence students’ learning experiences during fundamental nursing training. Previous CICARE research has predominantly focused on clinical staff or communication-related outcomes, leaving limited evidence regarding its integration with reflective learning and its potential influence on broader educational outcomes in undergraduate nursing students. Moreover, limited evidence is available on whether a structured communication framework, combined with reflective learning, can simultaneously support communication competence, empathy, professional identity, and clinical judgment within routine nursing skills education. This study integrates CICARE into routine nursing skills training and combines it with structured reflection. In doing so, it examines not only communication-related outcomes but also empathy, professional identity, and clinical judgment. Therefore, this study used a quasi-experimental design with an embedded qualitative component to examine the effects of a reflection-integrated CICARE communication program on communication competence, empathy, professional identity, and clinical judgment, while also exploring Intervention Group students’ learning experiences through reflective journals. The findings may provide practice-relevant evidence to inform communication-focused curriculum development in undergraduate nursing education.

## 2. Materials and Methods

### 2.1. Study Design

This study comprised a quasi-experimental quantitative component and an embedded qualitative component. The quantitative component employed a non-equivalent control group design, with pretest and posttest assessments of communication competence, empathy, and professional identity. Clinical judgment was assessed only after the intervention, and course performance was evaluated using the routine course assessment scheme. The qualitative component consisted of reflective journals collected from students in the Intervention Group to explore their experiences of the CICARE-based program and to provide contextual information for interpreting the quantitative findings. Because comparable qualitative data were not collected from the Control Group, the qualitative component was intended to complement the quantitative findings rather than to compare learning experiences between groups.

### 2.2. Participants and Setting

**Figure 1:**
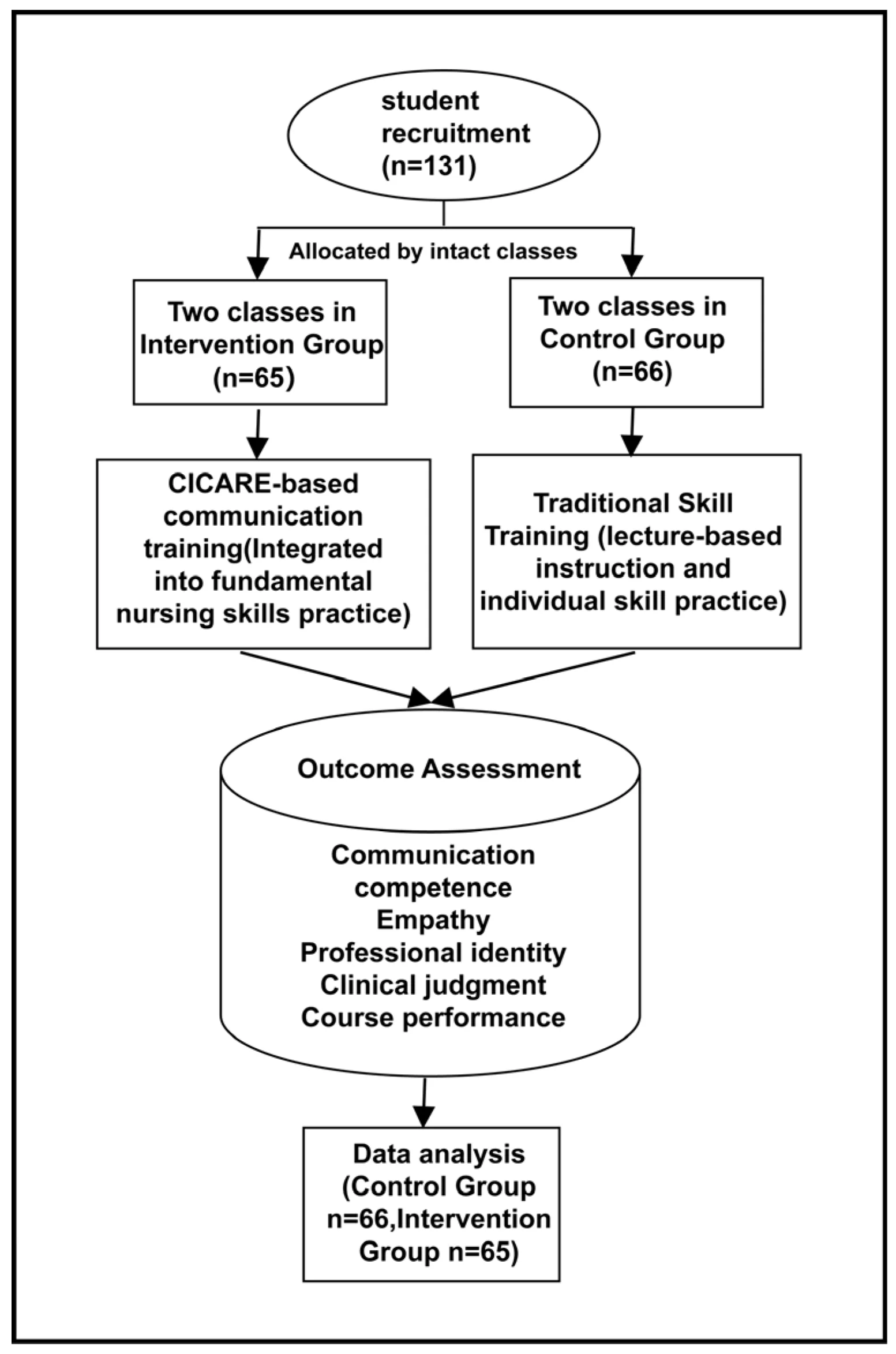
Flow Diagram of the Study Design and Intervention.

Undergraduate nursing students enrolled in a Bachelor of Science in Nursing program at a nursing college in China were recruited using convenience sampling from the 2023 cohort. All participants were second-year students taking the required Fundamentals of Nursing course during the study period. The inclusion criteria were enrollment in the Fundamentals of Nursing course, agreement to participate in the study, and completion of the required pretest and posttest assessments. Because the intervention was delivered within existing teaching units, individual randomization was not feasible, and allocation was therefore conducted at the intact-class level. The students had been assigned to four intact classes by the institution before the study began. For the present study, two intact classes were assigned to the Intervention Group and two to the Control Group according to the existing teaching arrangements. Individual students were not reassigned between classes. This approach could introduce selection bias and unmeasured class-level differences. To reduce this risk, all participants were recruited from the same academic cohort, were enrolled in the same required Fundamentals of Nursing course, and completed the study during the same teaching period. Baseline demographic characteristics and pre-intervention outcome measures were compared between groups to assess baseline equivalence. In addition, both groups were evaluated using the same course assessment criteria and scoring standards. A total of 131 students completed the study, including 65 students in the Intervention Group and 66 students in the Control Group. The Intervention Group received the CICARE-based communication program integrated into fundamental nursing skills training, whereas the Control Group received conventional skills training. Figure 1 presents the flow diagram of the study design and intervention. No statistically significant between-group differences were observed in the measured baseline characteristics.

### 2.3. Sample Size Considerations

Because the intervention was implemented in intact classes, the sample size was primarily determined by class enrollment. To assess whether the available sample was adequate, a sample size analysis was conducted using G*Power 3.1. The ANCOVA framework was selected because communication competence, empathy, and professional identity were measured before and after the intervention and the primary between-group analyses compared post-intervention scores while adjusting for the corresponding baseline values. A similar non-equivalent control-group nursing education study also used ANCOVA to evaluate post-intervention communication outcomes [23]. For an analysis of covariance with two groups, one baseline covariate, a two-sided α of 0.05, 80% power, and a medium effect size of f = 0.25, a minimum total sample of 128 participants was required. The medium effect size was used as a conventional planning value rather than being derived from the cited study. The final sample of 131 students exceeded this threshold, indicating that the available sample was adequate to detect a medium between-group effect under these assumptions.

### 2.4. Intervention

The intervention was conducted within the practical component of the Fundamentals of Nursing course. The course lasted 18 weeks and included 56 class periods. Most weekly sessions comprised three 40-min class periods, totaling 120 min, whereas two sessions comprised four class periods, totaling 160 min. Practical teaching was conducted in the nursing skills laboratory, with an approximate student-to-faculty ratio of 30:2 during skills training sessions.

Before implementation, faculty members participated in weekly 1.5-h training sessions and joint lesson-planning meetings to standardize the delivery of the CICARE-based teaching program. These activities focused on the CICARE workflow, communication scenarios, emotional cues used in role-play activities, teaching procedures, and evaluation criteria. The same core teaching sequence, communication scenarios, and evaluation standards were applied across the intervention classes to minimize instructor-related variation. During the 18-week intervention, the teaching team continued joint lesson-planning meetings before relevant sessions to review the CICARE workflow, communication scenarios, emotional cue prompts, teaching procedures, and evaluation criteria.

The teaching team developed a CICARE-based communication workflow tailored to fundamental nursing procedures, including vital signs measurement, oxygen administration, suctioning, intramuscular injection, and intravenous infusion. The six steps of the CICARE model, namely Connect, Introduce, Communicate, Ask, Respond, and Exit, were systematically integrated into nursing skills training. The intervention aimed to support students in developing structured nurse-patient communication behaviors and recognizing and responding to patients’ emotional cues.

Before class, students completed assigned readings based on clinical communication scenarios. During class, scenario-based role-play, peer practice, instructor demonstration, and feedback were used to support application of the CICARE communication process. In selected training activities, students assumed the role of patients during peer role-play exercises. These students were provided with brief scenario instructions and emotional cues to promote consistency in role enactment. After class, students watched CICARE demonstration videos and completed reflective journals documenting communication difficulties, emotional responses, and plans for improvement.

A scenario involving a 6-year-old pediatric patient requiring a subcutaneous injection was used as a teaching demonstration case. Table 1 provides an overview of the reflection-integrated CICARE communication program, including the major implementation phases, learning objectives, teaching strategies, procedures supporting implementation consistency, and evaluation approaches. A detailed example illustrating how the six CICARE steps were applied in the pediatric injection scenario is provided in Supplementary Table S1.

### 2.5. Control Condition

Students in the Control Group received conventional skills training for the Fundamentals of Nursing course. Teaching activities included theoretical instruction, instructor demonstration, student practice, and practical skills assessment. In contrast to the intervention condition, structured CICARE-based communication procedures, scenario-based emotional cue training, and reflective journal writing were not included in the Control Group instruction.

### 2.6. Outcome Measures

#### 2.6.1. Communication Competence

Communication competence was assessed using a Chinese nursing communication competence scale developed for nursing-related populations [24]. The scale evaluates students’ perceived ability to communicate effectively in interpersonal and clinical contexts. Higher scores indicate better communication competence. In the present sample, Cronbach’s α was 0.947 at baseline and 0.959 at post-intervention.

#### 2.6.2. Empathy

Empathy was assessed using the Chinese version of the Jefferson Scale of Empathy–Health Profession Students. The original Jefferson Scale of Empathy was developed by Hojat et al. to measure empathy among healthcare professionals in patient care contexts [25], and the Chinese version has demonstrated acceptable psychometric properties among health profession students [26]. Items were rated on a 7-point Likert scale, with higher scores indicating greater empathy. In the present sample, Cronbach’s α was 0.728 at baseline and 0.786 at post-intervention.

#### 2.6.3. Professional Identity

Professional identity was measured using the Nursing Professional Identity Scale for nursing students developed by Hao et al. [27]. The scale assesses nursing students’ recognition of the nursing profession, including professional value, professional emotion, self-efficacy, and professional commitment. Items are rated on a 5-point Likert scale, with higher scores indicating stronger professional identity. Reverse-coded items were recoded before the total score was calculated. In the present sample, Cronbach’s α was 0.862 at baseline and 0.889 at post-intervention.

#### 2.6.4. Clinical Judgment

Clinical judgment was assessed using an adapted Chinese version of the Lasater Clinical Judgment Rubric. The original Lasater Clinical Judgment Rubric was developed to evaluate nursing students’ clinical judgment in simulation-based learning [28], and its Chinese version has been validated among Chinese undergraduate nursing students [29]. The scale used in this study included 11 items covering four domains of clinical judgment: noticing, interpreting, responding, and reflecting. Items were rated on a 4-point Likert scale, with higher scores indicating greater perceived clinical judgment ability. Clinical judgment was assessed only after the intervention. In the present sample, Cronbach’s α was 0.959.

### 2.7. Course Performance Assessment

Course performance included both formative and summative assessment components. Formative assessment consisted of attendance, routine classroom performance, and assignment completion, whereas summative assessment consisted of a final practical skills examination. Both groups were assessed using the same evaluation criteria and scoring standards. Assessor blinding was not feasible because instructors were aware of group allocation; therefore, standardized assessment criteria and scoring procedures were applied to both groups.

Although communication competence was not evaluated as an independent scoring domain in the course assessment, communication-related behaviors were incorporated into the operational procedure checklist. These behaviors included greeting patients, verifying patient identity, explaining procedures, providing instructions, and offering closing information or expressions of thanks. The overall course score was calculated according to the routine course assessment scheme.

### 2.8. Qualitative Data Collection and Analysis

To explore students’ learning experiences with the CICARE-based communication program, reflective journal entries were collected only from students in the Intervention Group. The Control Group did not receive the CICARE-based communication program or structured reflective writing activities; therefore, reflective journals were not collected from the Control Group. The qualitative component was not intended to compare learning experiences between groups but to provide contextual information for interpreting the quantitative findings and to better understand how students experienced the intervention.

Students in the Intervention Group were asked to reflect on communication difficulties, their experiences of applying the CICARE communication model, emotional responses during the learning process, and plans for self-improvement. Example prompts are provided in Supplementary Table S1.

A total of 65 reflective journal entries were collected from the 65 students in the Intervention Group. All journal entries were anonymized before analysis. The reflective journals were analyzed using thematic analysis following the approach described by Braun and Clarke. Two researchers independently reviewed and coded the data. Initial codes were compared and discussed until agreement was reached. Themes were developed through repeated review and comparison of the reflective journals. An audit trail was maintained throughout the analytic process to enhance transparency and consistency. Disagreements were resolved through discussion with a third researcher when necessary. The qualitative findings were considered alongside the quantitative results as contextual information and were also used to inform future refinement of the teaching program.

### 2.9. Data Analysis

Quantitative data were analyzed using SPSS version 26.0. Continuous variables are presented as mean ± standard deviation, and categorical variables are presented as frequencies and percentages. Among the 131 students included in the final analysis, no missing values were identified for the primary quantitative outcomes; therefore, no data imputation was performed. Baseline characteristics were compared using independent-samples *t* tests or chi-square tests, as appropriate.

For outcomes measured both before and after the intervention, post-intervention scores were compared between groups using analysis of covariance, with group as the fixed factor and baseline score as the covariate. Adjusted mean differences, 95% confidence intervals, F values, *p* values, and partial η^2^ were reported.

For clinical judgment, which was assessed only after the intervention, independent-samples *t* tests were used to compare total and domain scores between groups. Cohen’s d was calculated as a measure of effect size. Course performance was also compared between groups using independent-samples *t* tests.

Pearson correlation analysis was used to examine associations among changes in communication competence, empathy, professional identity, and post-intervention clinical judgment. Change scores were calculated as post-intervention scores minus baseline scores. A two-sided *p* value of less than 0.05 was considered statistically significant.

### 2.10. Ethical Considerations

This study was approved by the Ethics Committee of the Clinical Medical College of Anhui Medical University (Approval No. LCYXY00010). Written informed consent was obtained from all participants before data collection. Participation in the research component was voluntary, and students were informed that participation in or withdrawal from the research would not affect their academic evaluation. All data were anonymized before analysis to protect participants’ privacy and confidentiality.

## 3. Results

### 3.1. Baseline Characteristics

A total of 131 undergraduate nursing students completed the study, including 65 students in the Intervention Group and 66 students in the Control Group. Baseline demographic characteristics and outcome measures were comparable between the two groups. No significant between-group differences were observed in sex distribution, communication competence, empathy, or professional identity before the intervention (all *p* > 0.05) (Table 2).

### 3.2. Effects of the Intervention on Communication Competence, Empathy, and Professional Identity

After adjustment for baseline scores using analysis of covariance, significant between-group differences were observed in communication competence and empathy (Table 3).

Students in the Intervention Group had significantly higher post-intervention communication competence scores than those in the Control Group (adjusted mean difference = 2.699, *p* = 0.041). Empathy was also significantly higher in the Intervention Group (adjusted mean difference = 3.796, *p* = 0.023). In contrast, although professional identity scores were higher in the Intervention Group, the between-group difference did not reach statistical significance (adjusted mean difference = 3.018, *p* = 0.073).

### 3.3. Effects of the Intervention on Clinical Judgment

Students in the Intervention Group achieved significantly higher total LCJR scores than those in the Control Group, corresponding to a moderate-to-large between-group effect (Cohen’s d = 0.72, *p* < 0.001) (Table 4). Domain-specific analyses showed significantly higher scores in the Intervention Group for noticing, interpreting, and reflecting, with moderate effect sizes. Although responding scores were also higher in the Intervention Group, the between-group difference did not reach statistical significance and the effect size was smaller (Cohen’s d = 0.34, *p* = 0.057).

### 3.4. Correlations Among Educational Outcomes

Positive correlations were observed among changes in communication competence, empathy, and professional identity, with correlation coefficients ranging from 0.436 to 0.514 (Table 5). These findings indicate that improvements in these communication- and affect-related outcomes tended to occur concurrently.

In contrast, post-intervention clinical judgment was not significantly correlated with changes in communication competence (r = 0.127), empathy (r = 0.073), or professional identity (r = 0.081). Thus, improvements in these outcomes were not directly associated with post-intervention clinical judgment scores in the present sample.

### 3.5. Comparison of Course Performance Between the Two Groups

The Intervention Group achieved significantly higher course performance than the Control Group across all assessment components (Table 6). Significant between-group differences were observed for formative assessment scores (*p* = 0.019), summative assessment scores (*p* < 0.001), and overall course scores (*p* < 0.001).

### 3.6. Thematic Analysis of Reflective Journals

After the course, 65 reflective journals were collected from the Intervention Group. Because the qualitative component of this study aimed to explore students’ experiences with the CICARE-based communication training, only journals from the Intervention Group were included in the thematic analysis. All journals were read and coded by the research team, and meaningful units were identified and synthesized into three final themes. The themes are presented below as descriptions of students’ experiences as expressed in their reflective journals.

#### 3.6.1. Theme 1: Communication as a Relational Component of Nursing Practice

Students frequently described communication as extending beyond the clear delivery of information to include listening, understanding patients’ concerns, and providing emotional support. Representative entries included: “I used to think communication simply meant explaining things clearly. After experiencing simulated nursing scenarios, I realized that real communication requires understanding patients’ worries, pain, and needs with genuine empathy.” Another student wrote, “This course taught me how to listen. I realized that even a look or a few encouraging words can make patients feel respected and understood.” A third student wrote, “Effective communication not only improves nursing efficiency but also helps reduce patients’ fear and anxiety, making them more willing to cooperate with treatment.” Across these entries, students emphasized the relational and emotional dimensions of nurse-patient communication and described listening, understanding, and emotional support as important aspects of nursing practice.

#### 3.6.2. Theme 2: Patient Perspective and Empathic Awareness

Students frequently described considering nursing situations from the patient’s perspective and paying attention to patients’ emotional experiences. One student noted, “When I played the role of a patient, I experienced for the first time the helplessness brought by illness and realized how essential nurses’ patience and caring attitudes are.” Another wrote, “While communicating as a nurse, I found that understanding the patient’s emotions and feelings was more important than simply explaining the treatment.” A third student commented, “This course made me realize that empathy is not just a phrase—it requires truly putting oneself in the patient’s position; only by understanding patients can we genuinely help them.” Students also described an intention to pay greater attention to emotional support, nonverbal communication, and individualized interaction in their future clinical learning and practice.

#### 3.6.3. Theme 3: Professional Reflection and Self-Directed Learning

Students’ reflections also referred to the professional meaning of nursing, self-awareness, and motivation for self-directed learning. Representative entries included: “This course made me more certain that I am suited for nursing, and I truly hope to become a nurse who brings warmth to patients,” “Writing reflective journals encouraged me to actively search for literature and learn communication strategies—I realized that active learning is far more effective than passive listening,” and “I discovered that self-reflection helps me identify areas for improvement, and I plan to continue using this learning approach in other courses.” Across these entries, students described awareness of professional values, their own learning needs, and areas for future improvement. These accounts represent experiences reported by students in the Intervention Group and are not interpreted as evidence of a between-group change in professional identity.

### 3.7. Integration of Quantitative and Qualitative Findings

Considering the quantitative and qualitative findings together, the reflective journals provided contextual information about how students in the Intervention Group described communication, empathy, and reflection. These accounts were broadly consistent with the higher quantitative scores in communication competence and empathy, but they do not constitute a between-group qualitative comparison. Students also described greater awareness of patients’ emotions and communication needs while noting difficulty translating this awareness into timely clinical action. This observation is compatible with the non-significant between-group difference in the responding domain, although the qualitative data cannot establish a causal explanation for the quantitative pattern. Table 7 presents these quantitative findings alongside the corresponding qualitative observations and cautious contextual interpretations.

## 4. Discussion

This study examined whether a reflection-integrated CICARE communication program could improve undergraduate nursing students’ communication-related learning outcomes when embedded in a Fundamentals of Nursing skills course. After adjustment for baseline scores, students in the Intervention Group had higher communication competence and empathy than those in the Control Group. The Intervention Group also achieved higher total clinical judgment scores and better course performance. However, the between-group difference in professional identity was not statistically significant, and the responding domain of clinical judgment also did not reach statistical significance. Overall, the findings suggest that the program may be more effective in supporting communication awareness, empathic understanding, cue recognition, and reflective thinking than in producing immediate changes in professional identity or action-oriented clinical responses.

The higher post-intervention empathy score in the Intervention Group is an important finding. In conventional skills training, students may focus primarily on completing procedures accurately and safely, while the patient’s emotional experience can easily become secondary. The CICARE-based program required students to ask about patients’ concerns, respond to emotional cues, and close the interaction in a respectful and reassuring manner. These steps may have helped students move beyond task completion and pay closer attention to the patient as a person. Role-play and scenario-based practice also allowed students to experience clinical procedures from both the nurse’s and the patient’s perspectives. This may help account for the higher empathy score observed in the Intervention Group. The reflective journals were consistent with this interpretation, as students frequently described a shift from “explaining clearly” to “listening,” “understanding patients’ worries,” and “providing emotional support.”

The present findings are broadly consistent with recent international studies showing that reflective writing and communication simulation can improve communication-related and empathic outcomes among nursing students [19,20]. However, the current study extends these findings by integrating structured communication practice and reflection within routine nursing skills training rather than delivering them as separate educational activities. The higher scores in noticing, interpreting, and reflecting are also consistent with previous simulation-based studies of clinical judgment [21,22]. In contrast, the non-significant between-group difference in responding suggests that structured communication and reflection alone may be insufficient to produce immediate changes in action-oriented clinical judgment.

Ding et al. evaluated an empathy-focused clinical education program in Chinese nursing students using the Jefferson Scale of Empathy–Health Professions Student, a clinical communication competence scale, and a professional identity scale; the intervention group had higher post-intervention empathy, communication, and professional identity scores than the control group [30]. The present findings were similar for empathy and communication competence, whereas professional identity did not differ significantly between groups. This difference may reflect variation in educational context and duration of exposure, as the prior intervention was delivered during a 10-month clinical internship whereas the present program was embedded in an 18-week skills course. For clinical judgment, Yang et al. used the Chinese LCJR in four classes of undergraduate nursing students and reported higher scores across noticing, interpreting, responding, and reflecting after simulation-based teaching [29]. In the present study, noticing, interpreting, and reflecting were higher in the Intervention Group, whereas responding did not reach statistical significance. This contrast suggests that action-oriented responding may require more intensive or dynamic simulation practice than was included in the present communication-focused course.

The clinical judgment findings should be interpreted in relation to the structure of the intervention. Students in the Intervention Group performed better in noticing, interpreting, and reflecting, while the difference in responding did not reach statistical significance. Beyond statistical significance, the magnitude of these differences is also educationally relevant. The between-group effect for total clinical judgment was moderate to large (Cohen’s d = 0.72), while the effects for noticing, interpreting, and reflecting were in the moderate range. In contrast, the responding domain showed a smaller effect (Cohen’s d = 0.34) and did not reach statistical significance. This pattern suggests that the intervention may have been more effective in strengthening students’ awareness of patient cues, interpretation of clinical information, and reflective thinking than in producing immediate changes in action-oriented clinical behavior. The CICARE process begins with observing the patient, establishing rapport, clarifying information, asking about needs, and responding to concerns. These activities may support students’ ability to notice relevant cues and interpret the patient’s situation. Reflective writing after practice may further strengthen students’ ability to review their communication behaviors and reconsider how they handled patient interactions. Responding, however, requires students not only to recognize and understand a clinical situation but also to prioritize competing information and translate judgment into timely action. These behaviors may require repeated exposure to dynamic clinical situations, high-fidelity simulation, and immediate performance-based feedback beyond what can be achieved through structured communication training within a single course. The absence of significant correlations between post-intervention clinical judgment and changes in communication competence, empathy, and professional identity also warrants consideration. One possible explanation is that these outcomes represent related but distinct dimensions of nursing competence and may develop through different educational mechanisms and at different rates. Communication competence and empathy primarily reflect interpersonal and affective development, whereas clinical judgment additionally depends on clinical knowledge, prioritization, situational interpretation, and decision-making under changing conditions. Furthermore, clinical judgment was measured only after the intervention, whereas the other outcomes were represented by pretest-to-posttest change scores. This difference in measurement structure may have limited the ability to detect meaningful associations. Therefore, the absence of statistically significant correlations should not necessarily be interpreted as evidence that these competencies are unrelated. Future longitudinal studies incorporating baseline and repeated measures of clinical judgment could better clarify the temporal and potentially interactive relationships among these outcomes.

The non-significant finding for professional identity also deserves careful discussion. Although the Intervention Group had a higher adjusted professional identity score than the Control Group, the between-group difference was not statistically significant. Therefore, this result should not be interpreted as evidence that the program directly improved professional identity. Professional identity is usually shaped through repeated professional socialization, clinical exposure, role modeling, and long-term internalization of nursing values. A single-course intervention may help students reflect on the meaning of nursing, but it may not be sufficient to produce a measurable change within one semester. In the reflective journals, some students described greater awareness of the professional value of nursing and stronger motivation for self-directed learning and future clinical practice. These accounts suggest engagement in professional reflection, while the measurable effect on professional identity remains uncertain.

The quantitative and qualitative components provided complementary perspectives on the findings. Quantitatively, the Intervention Group showed higher communication competence, empathy, total clinical judgment, and course performance. Qualitatively, students in the Intervention Group described communication as a relational process involving listening, attention to emotional needs, and consideration of the patient’s perspective. They also described reflection on their own communication behaviors and learning needs. These accounts are broadly consistent with the quantitative patterns observed for empathy and the cognitive-reflective components of clinical judgment and provide contextual insight into how students experienced the program. However, because comparable qualitative data were not collected from the Control Group, the reflective journals cannot establish that these experiences differed between groups or that they explain the observed quantitative effects. Similarly, students’ descriptions of difficulty translating awareness into timely clinical action provide useful context for the smaller and non-significant difference in responding but should not be interpreted as evidence of the mechanism underlying that quantitative result.

Another finding was that students in the Intervention Group achieved higher formative, summative, and overall course performance scores. This result may be related to the way the CICARE program was embedded into routine skills training rather than delivered as an isolated communication module. Students repeatedly practiced communication behaviors alongside technical procedures, including greeting patients, verifying identity, explaining procedures, giving instructions, and closing the interaction appropriately. Such integration may have helped students organize their procedural performance more clearly and present their skills in a more complete and patient-centered manner during assessments. However, because communication-related behaviors were incorporated into the operational checklist rather than assessed as an independent domain, this finding should be interpreted cautiously. Future studies could use a more detailed assessment rubric to distinguish technical performance, communication behaviors, and patient-centered responses.

### 4.1. Educational Implications

This study has several educational implications. First, structured communication training may be most useful when it is integrated into existing skills courses rather than added as a separate teaching activity. In this study, the CICARE framework was applied to common fundamental nursing procedures, making communication practice closely linked to students’ routine learning tasks. Second, reflection appears to be an important component of communication training. Reflective journals encouraged students to review their own difficulties, emotional responses, and plans for improvement, which may have supported deeper learning. Third, the findings suggest that communication education should not only aim to improve students’ verbal expression but should also support empathy, cue recognition, and reflective clinical thinking. These outcomes are especially relevant for undergraduate students who are transitioning from laboratory-based skills practice to clinical learning.

### 4.2. Limitations

Several limitations should be acknowledged. First, this study used a quasi-experimental design with non-random intact-class allocation. Because class-level randomization was not undertaken, selection bias and residual class-level confounding cannot be excluded despite baseline comparability between groups. Students in the Intervention and Control Groups were enrolled in the same institution and academic cohort, so informal exchange of learning experiences or communication strategies between classes could not be completely excluded; such contamination may have reduced the observed between-group differences. In addition, because outcome assessors were not blinded to group allocation, observer expectancy bias cannot be completely excluded. Second, the study was conducted in a single nursing college, and the findings may not be generalizable to students from other institutions or educational contexts. Third, communication competence, empathy, and professional identity were measured using self-report scales, which may be influenced by social desirability or students’ expectations of the course. Fourth, clinical judgment was assessed only after the intervention, so baseline-adjusted changes in clinical judgment could not be examined. Fifth, reflective journals were collected only from the Intervention Group. Therefore, the qualitative findings describe only the experiences of students who received the CICARE-based program and do not support between-group qualitative comparisons or causal attribution to the intervention. Finally, the study did not include long-term follow-up, and it remains unclear whether the observed between-group differences would persist during later clinical practice.

Overall, this study suggests that a reflection-integrated CICARE communication program may help undergraduate nursing students develop communication competence, empathy, and selected aspects of clinical judgment within fundamental nursing skills training. The findings also indicate that professional identity and action-oriented clinical responses may require longer and more intensive educational exposure. Although the findings should not be generalized beyond the present educational setting, they provide context-specific evidence that may inform replication studies and future multicenter evaluations of structured communication and reflective learning in nursing education. Future studies should consider randomized class-level allocation where feasible, multicenter designs, longer follow-up periods, more objective performance assessments, and qualitative data collection from both Intervention and Control Groups.

## 5. Conclusions

This study integrated CICARE-based communication training with structured reflection within fundamental nursing skills training for undergraduate nursing students. Students in the Intervention Group demonstrated higher communication competence, empathy, total clinical judgment, and course performance than those in the Control Group, whereas the between-group difference in professional identity was not statistically significant. In their reflective journals, students described greater patient-centered awareness, attention to patients’ emotional needs, and motivation for self-directed learning. These findings suggest that structured communication training combined with reflection may be a feasible approach to strengthening communication-focused nursing education. Future multicenter studies with larger and more diverse samples, longer follow-up periods, and more objective performance-based outcome measures are needed to determine whether the observed effects are sustained over time and generalizable across different nursing education settings. These findings may nevertheless inform replication studies and future multicenter research designed to confirm and extend the present findings.

## Figures and Tables

**Table 1:** Structure, Implementation, and Evaluation of the Reflection-Integrated CICARE Communication Program.

Phase	Learning Objectives	Main Activities	Teaching Strategies	Implementation Consistency/Monitoring	Evaluation
Faculty preparation	Standardize instructors’ understanding and delivery of the CICARE-based teaching program	Weekly 1.5-h faculty training before implementation; regular joint lesson-planning meetings; review of the CICARE workflow, communication scenarios, teaching procedures, and evaluation criteria	Faculty training and standardized instructional preparation	Teaching procedures, communication scenarios, and evaluation criteria were standardized across instructors.	Faculty preparation was completed before implementation; no separate faculty outcome assessment was performed
Pre-class preparation	Develop students’ preliminary understanding of clinical communication situations and patient needs	Assigned readings based on clinical communication scenarios	Self-directed learning and case-based preparation	Students in the Intervention Group followed the same CICARE-based teaching framework and preparatory requirements	No separate pre-class outcome assessment
In-class experiential learning	Apply the six CICARE steps during nursing procedures and strengthen students’ ability to recognize and respond to patients’ emotional cues	Scenario-based role-play, peer practice, patient-role enactment, instructor demonstration, and feedback	Experiential learning, role-play, peer learning, demonstration, and guided feedback	A standardized six-step CICARE workflow was used. Students assuming the patient role received brief scenario instructions and emotional cues to promote consistency in role enactment	Formative feedback was provided during practical training
Post-class reflection	Reflect on communication difficulties, emotional responses, application of CICARE, and areas for improvement	CICARE demonstration videos and structured reflective journal writing	Reflective learning and self-directed learning	Students reflected on common topics, including communication difficulties, CICARE application, emotional responses, and plans for self-improvement	Reflective journals from the Intervention Group were collected for thematic analysis
Outcome evaluation	Evaluate communication-related, affective, cognitive, and academic outcomes following the intervention	Assessment of communication competence, empathy, professional identity, clinical judgment, course performance, and students’ experiences documented in reflective journals	Quantitative and qualitative evaluation	The same outcome measures and course assessment criteria were applied as appropriate across groups	Communication competence, empathy, and professional identity: pretest and posttest; clinical judgment: post-intervention only; course performance: formative and summative assessment; reflective journals: thematic analysis

**Table 2:** Baseline Characteristics of Participants.

Variable	Control (*n* = 66)	Intervention (*n* = 65)	t/χ^2^	*p*
Female, *n* (%)	56 (84.8)	58 (89.2)	0.557	0.456
Emotional resonance	34.89 ± 4.79	34.78 ± 4.70	0.132	0.895
Perspective taking	28.71 ± 4.37	28.88 ± 3.03	0.251	0.802
Patient understanding	22.74 ± 4.49	22.80 ± 5.07	0.069	0.945
Empathy total score	86.35 ± 7.74	86.46 ± 6.29	0.092	0.927
Communication competence	54.38 ± 9.94	54.62 ± 7.04	0.157	0.875
Professional identity	58.62 ± 6.85	60.82 ± 8.23	1.486	0.140

Note. Values are presented as *n* (%) or mean ± standard deviation, as appropriate. χ^2^ = chi-square statistic.

**Table 3:** Baseline-Adjusted Effects on Communication Competence, Empathy, and Professional Identity.

Outcome	Control Group Adjusted Mean (SE)	Intervention Group Adjusted Mean (SE)	Adjusted Mean Difference	95% CI	F	*p*	Partial η^2^
Communication competence	55.836 (0.923)	58.536 (0.930)	2.699	0.108–5.291	4.247	0.041	0.032
Empathy	89.361 (1.160)	93.157 (1.169)	3.796	0.538–7.053	5.314	0.023	0.040
Professional identity	63.861 (1.170)	66.879 (1.179)	3.018	−0.283–6.319	3.273	0.073	0.025

Note. Adjusted means and mean differences were estimated using analysis of covariance with baseline scores as covariates. Adjusted group means are presented as estimated marginal means (standard errors). CI = confidence interval; η^2^ = partial eta squared.

**Table 4:** Between-Group Comparison of Clinical Judgment Scores.

Dimension	Control	Intervention	Mean Difference	95% CI	Cohen’s d	*p*
Noticing	7.79 ± 1.98	8.94 ± 1.62	1.15	0.53–1.78	0.64	<0.001
Interpreting	5.06 ± 1.37	5.74 ± 1.27	0.68	0.22–1.13	0.51	0.004
Responding	10.53 ± 2.70	11.38 ± 2.39	0.85	−0.02–1.75	0.34	0.057
Reflecting	4.67 ± 1.34	5.46 ± 1.15	0.79	0.36–1.23	0.64	<0.001
Total LCJR	27.20 ± 6.45	31.52 ± 5.50	4.33	2.26–6.40	0.72	<0.001

Note. LCJR = Lasater Clinical Judgment Rubric; CI = confidence interval. Clinical judgment was assessed only after the intervention. Cohen’s d represents the standardized between-group effect size.

**Table 5:** Pearson Correlations Among Changes in Communication Competence, Empathy, Professional Identity, and Post-Intervention Clinical Judgment.

Variable	1	2	3	4
1. Δ Communication Competence	-			
2. Δ Empathy	0.436 *	-		
3. Δ Professional Identity	0.472 *	0.514 *	-	
4. Clinical Judgment (Post-Intervention Total Score)	0.127	0.073	0.081	-

Note. Δ indicates the change score calculated as post-test minus pre-test. LCJR = Lasater Clinical Judgment Rubric. Clinical judgment was assessed only after the intervention; therefore, the post-intervention LCJR total score was used in the correlation analysis. Pearson correlation coefficients are presented. * *p* < 0.05.

**Table 6:** Course Performance Between the Two Groups.

Group/Statistic	Formative Assessment Score	Summative Assessment Score	Overall Score
Control Group	94.06 ± 2.31	92.65 ± 4.82	93.05 ± 3.56
Intervention Group	94.92 ± 1.78	95.25 ± 3.10	95.15 ± 2.33
t	2.368	3.658	3.984
*p*	0.019	<0.001	<0.001

Note. Formative assessment included attendance, routine classroom performance, and assignment completion. Summative assessment consisted of the final practical skills examination.

**Table 7:** Joint Display of Quantitative and Qualitative Findings.

Quantitative Finding	Qualitative Observation	Contextual Interpretation
Empathy was higher in the Intervention Group	Students described considering procedures from the patient’s perspective and paying greater attention to emotional needs	These observations are consistent with the quantitative empathy finding and provide contextual information, but they do not establish causality
Total LCJR, noticing, interpreting, and reflecting scores were higher in the Intervention Group	Students described greater attention to emotional and situational cues and reflection on their own communication behaviors	These observations provide context for the quantitative pattern in the cognitive and reflective aspects of clinical judgment but do not establish a mechanism of effect
The between-group difference in responding was not statistically significant	Some students described difficulty translating awareness of patients’ needs into appropriate or timely action	The qualitative accounts are compatible with the smaller quantitative difference in responding but should not be interpreted as a causal explanation
Professional identity did not differ significantly between groups	Some students described professional reflection, awareness of nursing values, and motivation for self-directed learning	These accounts describe professional reflection among Intervention Group students but do not demonstrate an intervention-related improvement in professional identity

Note. LCJR = Lasater Clinical Judgment Rubric. Reflective journals were collected only from the Intervention Group; therefore, the qualitative observations provide contextual information and cannot establish qualitative between-group differences or causal mechanisms.

## Data Availability

The data supporting the findings of this study are available from the corresponding author upon reasonable request due to privacy and ethical restrictions.

## Supplementary Materials

The following supporting information can be downloaded at: https://www.mdpi.com/article/10.3390/nursrep16090296/s1, Table S1: Scenario-based examples of CICARE-based teaching in basic nursing skills training.

## Public Involvement Statement

No public involvement in any aspect of this research.

## Guidelines and Standards Statement

This manuscript was drafted with reference to relevant reporting guidance for quasi-experimental and mixed-methods research.
