# Supplementary material for: Effects of a Reflection-Integrated CICARE Program on Empathy and Clinical Judgment in Nursing Students: A Quasi-Experimental Study
Source: Nurs Rep. 2026 Aug 24;16(9):296. doi: 10.3390/nursrep16090296 (PMC13610859; doi:10.3390/nursrep16090296)
Supplement: Supplementary file 1 [file nursrep-16-00296-s001.zip › nursrep-4459016-supplementary.pdf]

Supplementary Material

Table S1. Scenario-based examples of CICARE-based teaching in basic nursing skills training

CICARE step	Clinical scenario	Example of teaching implementation
**Connect**	Initial contact with a pediatric patient and caregiver	Students are guided to establish rapport by observing the child's facial expressions, body posture, and caregiver's emotional state. A calm tone, child-friendly language, and appropriate nonverbal communication (e.g., eye-level positioning, gentle posture) are used to reduce anxiety and build trust.
**Introduce**	Beginning of interaction before the procedure	Students are guided to introduce their professional role and explain the purpose of the interaction using simple, age-appropriate language. The procedure and expected process are briefly described to reduce uncertainty for both the child and caregiver.
**Communicate**	Explaining the nursing procedure	Students are guided to explain the procedure step by step, avoiding technical terminology. Expected sensations are described clearly, and reassurance is provided. Visual aids or demonstrations may be used to enhance understanding and reduce fear.
**Ask**	Assessing patient and	Students are guided to use open-ended questions to

	caregiver needs	explore concerns and preferences. The child is offered simple choices (e.g., selecting an arm or distraction method) to enhance perceived control and cooperation.
**Respond**	Addressing emotional responses during care	Students are guided to identify emotional cues such as fear or tension and respond with empathy and normalization. Reassurance is provided to promote emotional comfort and cooperation during the procedure.
**Exit**	Completion of the procedure	Students are guided to evaluate patient comfort, provide positive feedback, explain post-procedure care, and conclude the interaction with supportive language to reinforce a positive care experience.
